# In Memoriam, Herbert Mickle, M. D., C. M.

**Published:** 1904-02

**Authors:** W. Scott Renner


### In Memoriam, Herbert Mickle, M. D., C. M.

BY W. SCOTT RENNER, M. D.

Dr. Herbert Mickle, formerly of Buffalo, died at Asheville, N. C., May 22, 1903, of tuberculosis of the lungs, aged 43 years. Dr. Mickle's ill health commenced in an attack of pleurisy which he contracted about 12 or 13 years ago, in the devoted attention to his profession. From that time until the time of his death, he never enjoyed the robust health for which he was admired before that illness. He was born near Guelph, Canada, of Scotch-Canadian parentage and received his academic education at Upper Canada College. He studied medicine at Trinity College, from which institution he received the degree of M. D., C. M., in 1881; after which he went to London, where he devoted himself to the study of surgery and became a member of the Royal College of Surgeons, England, and a licentiate of the Royal College of Physicians, London, in 1883.

Directly after this, Dr. Mickle came to Buffalo and established himself as a surgeon. He was appointed surgeon to the Emergency Hospital, which was at that time located on Michigan Street. Dr. Mickle soon became prominent in the faculty of the Niagara University, in which institution he was appointed in 1885, assistant in surgery; in 1886, lecturer on pathology; in 1888, professor of descriptive and surgical anatomy; in 1891, professor of principles and practice of surgery; in 1896, professor of practice of surgery and clinical surgery, which position he retained until the amalgamation of the Buffalo and Niagara Medical Colleges in 1898, when he became clinical professor in surgery, in the medical department of the University of Buffalo.

Dr. Mickle was recognised as an able teacher of surgery and his ability to teach in this department was supported by a thorough knowledge of anatomy. He was for many years one of the attending surgeons at the Sisters of Charity Hospital and was, until he left Buffalo, one of the consulting surgeons of the

Buffalo General Hospital. He was an able and conservative surgeon. Few of his colleagues were acquainted with his knowledge of the diagnosis of internal diseases, and his ability in this line gave him a prominent place as an insurance examiner. Discouraged and broken down in health, Dr. Mickle tried another field of labor, but he was then, already doomed to die of that dreadful disease, tuberculosis.

Personally, Dr. Mickle was a gentleman of refinement, very popular in the circles of his acquaintanceship and held in the highest esteem by all who knew him for his attractive social qualities and his genial manner.

